# Clinical Outcomes and Inflammatory Response to the Enhanced Recovery After Surgery (ERAS) Protocol in Adolescent Idiopathic Scoliosis Surgery: An Observational Study

**DOI:** 10.3390/ijms26083723

**Published:** 2025-04-15

**Authors:** Francesca Salamanna, Giuseppe Tedesco, Maria Sartori, Deyanira Contartese, Emanuela Asunis, Chiara Cini, Francesca Veronesi, Konstantinos Martikos, Milena Fini, Gianluca Giavaresi, Alessandro Gasbarrini

**Affiliations:** 1 Surgical Sciences and Technologies, IRCCS Istituto Ortopedico Rizzoli, Via di Barbiano 1/10, 40136 Bologna, Italy; francesca.salamanna@ior.it (F.S.); maria.sartori@ior.it (M.S.); francesca.veronesi@ior.it (F.V.); gianluca.giavaresi@ior.it (G.G.); 2 Department of Spine Surgery, IRCCS Istituto Ortopedico Rizzoli, Via Pupilli 1, 40136 Bologna, Italy; giuseppe.tedesco@ior.it (G.T.); asunis.emanuela@gmail.com (E.A.); chiara.cini@ior.it (C.C.); konstantinos.martikos@ior.it (K.M.); alessandro.gasbarrini@ior.it (A.G.); 3 Scientific Direction, IRCCS Istituto Ortopedico Rizzoli, Via di Barbiano 1/10, 40136 Bologna, Italy; milena.fini@ior.it; 4 Department of Biomedical and Neuromotor Sciences, Alma Mater Studiorum University of Bologna, 40126 Bologna, Italy

**Keywords:** enhanced recovery after surgery, adolescent idiopathic scoliosis, clinical outcomes, inflammatory response, observational study

## Abstract

Limited data exist on the clinical outcomes and inflammatory response of Enhanced Recovery After Surgery (ERAS) protocols in adolescent idiopathic scoliosis (AIS) patients undergoing posterior spinal fusion (PSF). This study evaluates ERAS’s impact by analyzing blood parameters and serum protein, surgical, and radiological parameters to assess safety, feasibility, and clinical outcomes. Between July 2021 and July 2022, 30 AIS patients underwent PSF with standardized pain management, high-dose tranexamic acid, early mobilization, and reduced opioid use. Blood tests and serum markers (IL-6, IL-1β, IL-1α, IL-10, TNF-α, IL-8, PGE2) were measured preoperatively and on postoperative day 2. Pain levels (VAS) were recorded preoperatively, on postoperative days 1 and 2, and at discharge. Results showed increased postoperative white blood cell counts, reduced hemoglobin and hematocrit, and elevated C-reactive protein levels. IL-6 was the only inflammatory marker significantly elevated, indicating a controlled inflammatory response. Pain peaked on day 1 but significantly decreased by discharge, confirming the effectiveness of multimodal analgesia. The average hospital stay was 6.97 ± 2.03 days, with low rehospitalization (6.66%) and manageable complications (20%). In conclusion, ERAS effectively optimizes AIS patient recovery, stabilizing pain, reducing complications, and improving perioperative care.

## 1. Introduction

Scoliosis stands as the predominant back deformity among the pediatric demographic, characterized by a lateral curvature exceeding 10 degrees on the coronal plane of a standing radiograph [1]. The extent of deviation is assessed via the measurement of the Cobb angle, defined as the angle formed by the most tilted vertebral bodies [1,2]. While various factors can contribute to scoliosis, approximately 80% of cases manifest as idiopathic [1,2]. Adolescent idiopathic scoliosis (AIS) constitutes the most prevalent form of idiopathic scoliosis, impacting around 3% of individuals aged between 10 and 18 years [1]. Although the precise etiology of AIS remains elusive, several contributing factors have been identified [2]. Notably, genetics emerge as a significant risk determinant in AIS development. Moreover, gender significantly influences AIS, with females being seven times more likely to require surgery, while males often develop more rigid curves with a higher risk of progression [1]. Treatment options for AIS depend on age, curve severity, and skeletal maturity, including surgical and non-surgical approaches [3]. When the curve exceeds 50 degrees, posterior spinal fusion (PSF) is necessary for stabilization. Managing AIS patients undergoing PSF is challenging, particularly regarding postoperative pain control and hospitalization duration. While opioids remain the primary postoperative analgesic, alternatives such as epidural analgesia, intrathecal opioids, ketamine, and gabapentinoids are also used [4], though achieving optimal pain control remains difficult.

In this context, Enhanced Recovery After Surgery (ERAS) protocols have emerged as a comprehensive approach to optimizing postoperative pain control while minimizing opioid reliance [5]. ERAS encompasses a multimodal strategy that integrates pre-, intra-, and postoperative measures to enhance recovery and reduce complications [5]. Preoperatively, patient education, nutritional optimization, and preemptive analgesia help set the foundation for improved outcomes. Intraoperatively, standardized anesthetic techniques, fluid management, and blood-sparing strategies contribute to hemodynamic stability and reduced surgical stress. Postoperatively, ERAS prioritizes multimodal analgesia—such as NSAIDs, acetaminophen, regional anesthesia, and adjunctive agents like gabapentinoids—to enhance pain relief, reduce opioid-related side effects, and facilitate early mobilization [5,6]. While ERAS is well-established in adult orthopedic surgeries, its application in pediatric surgery remains limited, and studies on its use in AIS patients undergoing PSF are scarce, particularly regarding its effects on inflammatory response or blood parameters [6,7,8,9]. While the inflammatory response is essential for wound healing and infection resistance, it can also exacerbate pain, fatigue, and sleep disturbances [10,11]. In response to surgical injury, the cellular mechanism involves the activation of neutrophils and macrophages within the innate immune system through the secretion of proinflammatory cytokines such as tumor necrosis factor (TNF) alpha and interleukins (ILs), notably IL-1 and IL-6 [12,13]. Proinflammatory cytokines modulate the levels of circulating acute phase proteins, including C-reactive protein (CRP), albumin, ferritin, transferrin, and fibrinogen [14]. Indeed, it has been observed that concentrations of circulating acute phase proteins and cytokines correlate with the magnitude of the stress response, referred to as the systemic inflammatory response (SIR) to surgery [15]. Additionally, CRP and IL-6 have been also identified as having the strongest association with the extent of surgical injury. Despite the acknowledgment that spine surgery elicits an augmented postoperative SIR, the influence of ERAS protocols on SIR in patients undergoing spinal fusion for AIS has yet to be investigated.

The objective of this observational study is to evaluate clinical outcomes and inflammatory response in AIS patients undergoing surgery with the ERAS protocol. The study aims to assess the feasibility and safety of this approach while providing preliminary data on postoperative recovery and inflammation control for future comparative studies.

## 2. Results

### 2.1. Demographic and Clinical Characteristics

A total of 30 AIS patients who underwent PSF were treated with ERAS. There are four male patients and 26 female patients, with a total average age of 15.33 ± 1.75. The demographic characteristics of the patients are shown in Table 1. Regarding preoperative Lenke classification for AIS of curve type, among the main curve types, Type 1 was the most frequently observed, accounting for 38.1% of cases, indicating its predominance in the population studied. Regarding the sagittal modifier, the (+) symbol, indicative of hyperkyphosis, was the most common, present in 42.9% of cases. Finally, as for the lumbar modifier, an equal distribution was observed between Types A and C, each representing 14.3% of cases, while a significant proportion of entries remained unspecified.

Several blood parameters showed significant changes in the postoperative period compared to preoperative values (Table 2). Specifically, the white blood cell count increased significantly from 6.69 ± 0.95 × 10^9^/L preoperatively to 11.72 ± 3.23 × 10^9^/L postoperatively. Conversely, the red blood cell count and hemoglobin levels showed a marked reduction, decreasing from 4.74 ± 0.37 × 10^12^/L to 3.19 ± 0.46 × 10^12^/L, and from 13.40 ± 1.42 g/dL to 9.17 ± 1.29 g/dL, respectively. Hematocrit levels also significantly decreased, dropping from 40.30 ± 2.71% to 27.23 ± 3.75%, indicating a substantial postoperative change. Regarding white blood cell differentials, lymphocytes significantly decreased from 2.22 ± 0.49 × 10^9^/L to 1.28 ± 0.46 × 10^9^/L, while neutrophils increased from 3.76 ± 0.95 × 10^9^/L to 9.12 ± 2.57 × 10^9^/L. Additionally, eosinophil levels significantly dropped from 0.21 ± 0.12 × 10^9^/L to 0.05 ± 0.05 × 10^9^/L. Among coagulation parameters, the prothrombin activity index (INR) and activated partial thromboplastin time (aPTT) ratio significantly increased postoperatively, from 1.08 ± 0.06 to 1.25 ± 0.09, and from 1.15 ± 0.12 to 1.36 ± 0.13, respectively, suggesting an alteration in coagulation balance. Similarly, CRP, a marker of inflammation, showed a marked increase, rising from 0.16 ± 0.25 mg/dL to 10.88 ± 4.62 mg/dL, confirming a systemic inflammatory response in the postoperative period. Finally, a significant increase in glucose levels was observed, from 86.53 ± 10.14 mg/dL to 107.00 ± 7.75 mg/dL, along with a decrease in creatinine levels, from 0.66 ± 0.10 mg/dL to 0.56 ± 0.16 mg/dL, the latter potentially linked to changes in postoperative fluid balance.

Of all the blood parameters analyzed, only white blood cells, hemoglobin, hematocrit, neutrophils, prothrombin activity ratio, prothrombin activity INR, aPTT ratio, CRP, and creatinine were found to fall outside the reference range. The remaining parameters showed significant differences between preoperative and postoperative values but remained within the reference range.

### 2.2. Radiological and Surgical Characteristics of ERAS Patients

**Figure 1:**
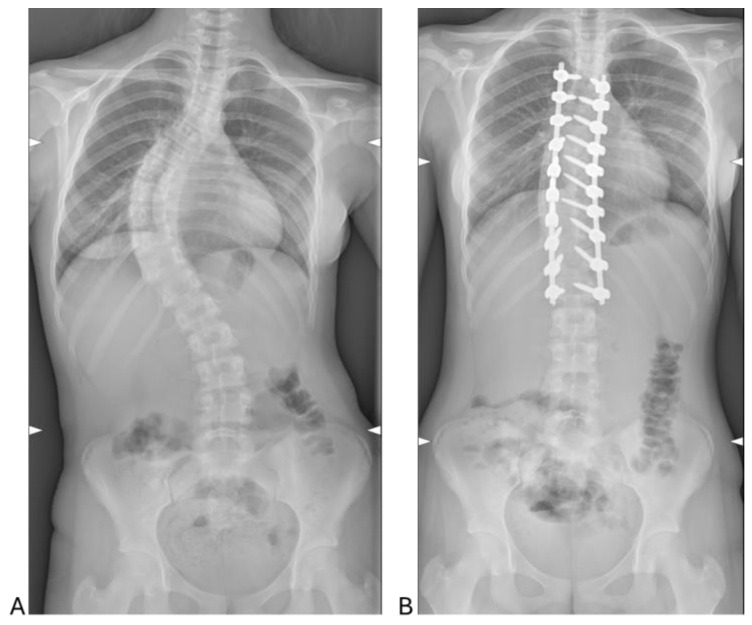
Pre- (**A**) and postoperative (**B**) radiographs of representative case with 13-year-old female patient. (**A**) Preoperative standing Cobb angle of 64° and (**B**) postoperative standing Cobb angle of 14°.

The analysis of radiographic outcomes showed a significant reduction in Cobb angles following surgery (Figure 1). Specifically, the lumbar Cobb angle decreased from 51.78 ± 12.90° preoperatively to 16.50 ± 3.82° postoperatively, reflecting a substantial correction of lumbar curvature. Similarly, the thoracic Cobb angle improved significantly, reducing from 61.82 ± 14.59° preoperatively to 14.33 ± 7.24° postoperatively. These results demonstrate the effectiveness of the surgical intervention in achieving marked corrections of both thoracic and lumbar spinal curvatures.

**Figure 2:**
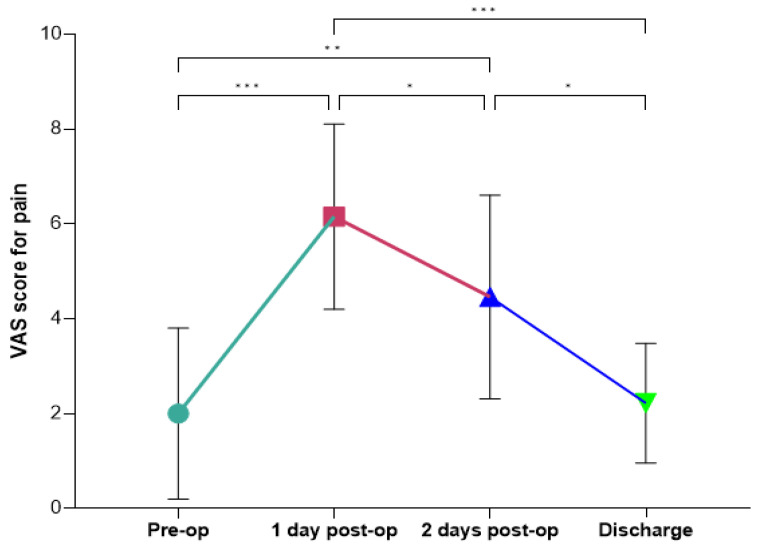
Plot of VAS score evaluated preoperatively, 1 and 2 days after surgery and at discharge. (*) *p*  <  0.05, (**) *p*  <  0.005, (***) *p*  <  0.0005.

Figure 2 shows the pain intensity scores, measured using the Visual Analog Scale (VAS), across four time points: preoperative (Pre-op), 1 day postoperative (1-day post-op), 2 days postoperative (2-days post-op), and discharge. Pain levels were mild preoperatively. Following surgery, there was a significant increase in pain, peaking on the first postoperative day. By the second postoperative day, pain intensity began to decline, and this trend continued until discharge, where pain levels returned to values comparable to the preoperative period. Statistical analysis revealed significant differences in pain scores between most time points (Figure 2). Pain intensity increased significantly from the preoperative period to the first postoperative day (*p* < 0.0001). A subsequent reduction in pain was observed between the first and second postoperative days (*p* < 0.05), as well as between the second postoperative day and discharge (*p* < 0.05). However, no significant differences were detected between the preoperative pain levels and those at discharge, indicating effective pain management and recovery over the postoperative period. These findings highlight the expected peak in pain shortly after surgery, followed by a steady decrease, ultimately returning to baseline levels by the time of discharge.

The analysis of outcomes related to the ERAS protocol provided valuable insights into perioperative management and patient recovery (Table 3). The average duration of surgery was 3.50 ± 0.90 h, indicating a relatively efficient operative time. Intraoperative blood transfusions were required in 20% of patients, reflecting the potential need for transfusion in a subset of cases despite the use of blood-sparing strategies commonly integrated within the ERAS framework.

The average length of stay (LOS) was 6.97 ± 2.03 days, demonstrating that the ERAS protocol facilitated relatively early discharge while maintaining safe recovery standards. The rate of re-hospitalization was 6.66%, suggesting a low incidence of postoperative complications necessitating readmission. Postoperative manageable complications (skin rush, diarrhea, pain in the iliac fossa, pain at the incision site) were observed in 20% of patients, highlighting the need for continued optimization of perioperative care to further minimize adverse events. These findings underscore the overall effectiveness of the ERAS protocol while identifying areas for potential improvement.

### 2.3. Pro-Inflammatory Parameters

**Figure 3:**
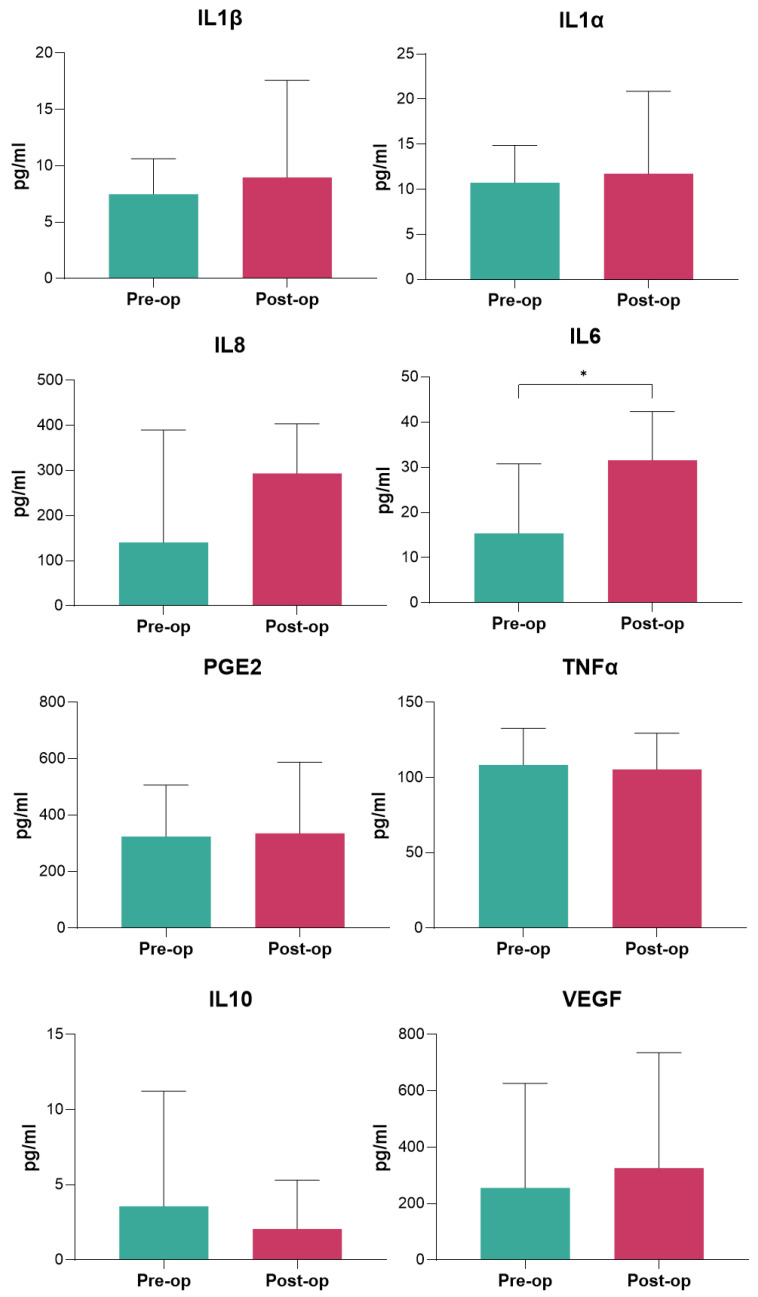
ELISA release of IL-1β, IL-1α, IL-6, IL-8, IL-10, TNF-α, and PGE2 pre-operatively and 2 days after surgery. Student’s *t*-test among pre-operative and post-operative time. Results were expressed as mean ± standard deviation (SD). IL6: pre-op versus post-op, (*) *p*  <  0.05.

Serum analyses of IL-1β, IL-1α, IL-6, IL-8, IL-10, TNF-α, and Prostaglandin E2 (PGE2) were conducted to evaluate differences in expression levels associated with the surgical procedure. Among these cytokines, a significant difference was observed in the expression of IL-6. In detail, the analysis revealed that pre-operative serum aliquots displayed significantly lower levels of IL-6 (*p* < 0.05) compared to post-operative serum aliquots, suggesting an increase in this inflammatory marker following surgery (Figure 3).

## 3. Discussion

This study provides valuable insights into the outcomes of 30 AIS patients treated with the ERAS protocol during PSF. The findings span demographic patterns, hematologic and inflammatory responses, radiographic corrections, and perioperative outcomes, offering a comprehensive evaluation of the protocol’s effectiveness.

The patient cohort, comprising predominantly females (26 out of 30), aligns with established epidemiological trends indicating a higher prevalence of AIS in females [16]. The average age of 15.33 ± 1.75 years is consistent with the typical timing for surgical intervention in this condition [17]. The predominance of Lenke Type 1 curves (38.1%) highlights thoracic scoliosis as the most common presentation in AIS, while the sagittal (+) modifier, associated with hyperkyphosis, was observed in 42.9% of cases. This reflects the diverse spinal deformities that characterize AIS, emphasizing the need for tailored surgical and perioperative management approaches [17]. In this context, the study demonstrates the efficacy of PSF in achieving significant radiographic corrections. Postoperative reductions in lumbar and thoracic Cobb angles, from 51.78° and 61.82° preoperatively to 16.50° and 14.33°, respectively, confirm the surgical technique’s ability to restore spinal alignment effectively.

Despite the surgical results and the use of the ERAS protocol, the surgery-induced physiological stress was reflected in significant changes in hematologic parameters. Postoperatively, white blood cell counts increased markedly, indicating an acute inflammatory response, while hemoglobin, hematocrit, and red blood cell levels decreased, likely due to intraoperative blood loss and fluid shifts [18]. The elevation in neutrophil counts, alongside reduced lymphocyte and eosinophil levels, reflects the activation of the innate immune response typical of the immediate postoperative period [19]. The rise in CRP levels further underscores the systemic inflammatory response, consistent with findings in similar surgical settings [20]. Notably, alterations in coagulation parameters, including increased prothrombin activity INR and aPTT ratios, suggest shifts in coagulation dynamics [21].

Among serum inflammatory markers, IL-6 displayed a significant postoperative increase, highlighting its role as a key mediator of the inflammatory response to surgical trauma [22]. However, elevated IL-6 levels are also typically associated with wound healing, immune modulation, and activation of the acute-phase response [22]. In spine surgery, postoperative IL-6 levels often rise 10- to 100-fold, peaking within the first 24–48 h, with values commonly ranging from 50–200 pg/mL depending on surgical complexity and patient factors [22]. Thus, in this study the observed increase in IL-6 is consistent with these norms, confirming the activation of a physiological inflammatory cascade. Interestingly, other cytokines, including IL-1β, IL-1α, IL-8, IL-10, TNF-α, and PGE2, did not show significant changes, suggesting a limited inflammatory response beyond IL-6 upregulation when employing the ERAS protocol. The absence of changes in anti-inflammatory cytokines such as IL-10 and the lack of a shift towards excessive immunosuppression further suggest that the ERAS protocol successfully maintained the inflammatory balance within a controlled range. This regulated response may contribute to preventing adverse outcomes, such as delayed recovery or infections.

Among the inflammatory serum markers analyzed, IL-6 was the only cytokine that demonstrated a significant postoperative increase, consistent with its well-documented role as a primary mediator of the acute inflammatory response to surgical trauma [22]. The lack of significant changes in other pro-inflammatory markers such as IL-1β, IL-1α, IL-8, TNF-α, and PGE2 suggests that the ERAS protocol may contribute to modulating excessive systemic inflammation, thereby preventing an exaggerated cytokine response. A possible explanation for the selective elevation of IL-6 could be its role as an early and transient marker of surgical trauma, peaking within the first 24–48 h before gradually declining [22]. Differently, IL-1β and TNF-α often rise earlier but may normalize more quickly, while IL-10, an anti-inflammatory cytokine, may peak later as part of the resolution phase of inflammation [22,23]. The absence of significant changes in IL-10 in our study suggests that the ERAS protocol may have helped maintain a balanced inflammatory state without excessive immunosuppression, which could otherwise predispose patients to infections or impaired healing [23,24]. Future studies with more frequent sampling at multiple perioperative time points (e.g., preoperatively, immediately postoperatively, at 6, 12, 24, and 72 h) could provide a more dynamic and complete profile of the inflammatory response. Additionally, assessing longer-term cytokine trends could help determine whether ERAS influences the resolution of inflammation and its impact on recovery outcomes.

In this study, the ERAS protocol also proved effective in optimizing perioperative care—with an average surgical duration of 3.50 ± 0.90 h and an LOS of 6.97 ± 2.03 days—and the study also revealed a low re-hospitalization rate (6.66%) and a manageable complication rate (20%), further reinforcing the protocol’s safety and reliability. In addition, although TXA was part of the ERAS protocol, our study found an intraoperative blood transfusion rate of 20%. It is important to note, however, that in AIS patients undergoing PSF without specific blood-sparing protocols, such as TXA, transfusion rates typically range from approximately 30% to 60%, depending on the severity of the scoliosis and the extent of the surgery [25,26]. Therefore, our study showed a significant reduction in the rate of transfusions compared to studies without specific blood-sparing protocols. However, to further improve blood-sparing outcomes, several potential strategies can be proposed: (1) Preoperative Anemia Management: optimizing hemoglobin levels preoperatively through iron supplementation, erythropoiesis-stimulating agents, or other interventions to address anemia may help reduce the need for transfusions during surgery; (2) Intraoperative Blood Management: implementing a more aggressive intraoperative hemodynamic optimization protocol could potentially reduce blood loss and minimize the need for transfusions; and finally (3) Cell Salvage Technology: The incorporation of intraoperative blood salvage, especially during high-blood-loss surgeries like PSF, could provide an additional strategy to minimize the need for allogenic blood transfusions [27]. 

Ensuring the patient’s recovery and improving their quality of life after spine surgery for AIS requires effective pain management [28]. In our cohort, the intensity of pain followed a predictable pattern, with a peak occurring on the first postoperative day and a gradual decline in the days that followed. This trajectory aligns with previous ERAS and non-ERAS studies that describe the immediate postoperative period as the most critical for pain management due to the physiological response to surgical trauma [29,30]. The observed significant increase in pain intensity from the preoperative period to the first postoperative day underscores the importance of proactive pain management strategies immediately following surgery. The peak in pain on day one is consistent with the inflammatory response and tissue repair processes that typically occur after surgical intervention. This reinforces the need for effective multimodal analgesic protocols to mitigate patient discomfort during this critical phase. The progressive decline in pain from the second postoperative day to discharge, with significant reductions observed between day one and day two, as well as between day two and discharge, reflects the natural resolution of the acute inflammatory phase and the effectiveness of postoperative pain management strategies within the ERAS protocol [29,30]. This effective pain control is clinically significant because it likely contributed to early mobilization, enhanced recovery, and improved functional outcomes. Additionally, returning to preoperative pain levels by discharge highlights the success of multimodal analgesia in mitigating discomfort and facilitating recovery, which is essential for preventing complications such as muscle deconditioning, respiratory issues, and thromboembolic events [30,31]. These outcomes ultimately support the efficacy of the ERAS protocol in optimizing recovery and promoting patient well-being after surgery.

This study has several limitations that should be acknowledged. First, the absence of a control group following a conventional postoperative recovery pathway limits our ability to definitively determine the superiority of ERAS over standard management. While our findings suggest improved pain control, reduced complications, and feasible implementation of ERAS in AIS patients undergoing PSF, a direct comparison with a non-ERAS cohort would strengthen these conclusions. Future randomized or controlled studies are necessary to validate these observations and establish ERAS as the optimal perioperative strategy in this population. Second, the study was conducted at a single institution with a relatively small sample size. Although our cohort reflects real-world clinical practice, the limited number of patients may affect the statistical power and generalizability of the results. Larger, multicenter studies would provide more robust data and account for potential variations in surgical techniques, perioperative care, and institutional protocols. Lastly, while we analyzed a comprehensive set of hematologic and inflammatory markers, additional long-term follow-up data are needed to assess the sustained impact of ERAS on patient recovery and functional outcomes.

Despite the encouraging findings demonstrated by the ERAS protocol in our patient cohort, several limitations warrant consideration. The lack of a control group, the small sample size and the single-center design of the study may limit the generalizability of the results. Larger, multicenter studies are mandatory to validate these findings and refine ERAS protocols further. Additionally, the potential of inflammatory markers, particularly IL-6, in predicting recovery trajectories and complications deserves further exploration. Blood management strategies should also be optimized to reduce transfusion rates, enhancing patient outcomes.

Despite these limitations, this study highlights the efficacy of the ERAS protocol in optimizing perioperative management for AIS patients undergoing PSF. The protocol’s success is evident in stable pain outcomes, a low complication rate, and streamlined recovery, reinforcing its reliability and safety. Notably, the findings underscore the importance of monitoring hematologic and inflammatory markers, particularly IL-6, as key indicators of surgical stress and recovery progression. However, further refinement is needed, particularly in blood-sparing strategies. Finally, a deeper exploration of cytokine dynamics and their interaction with perioperative protocols could pave the way for more personalized recovery strategies. By continuously evolving and incorporating these advancements, the ERAS protocol has the potential to further enhance patient safety, accelerate postoperative recovery, and solidify its position as the gold standard in AIS surgery.

Innovations in minimizing surgical blood loss, such as enhanced coagulation management, the use of innovative antifibrinolytic agents, and advanced surgical techniques, will be pivotal in improving patient outcomes. Moreover, continued research into cytokine dynamics and their interplay with perioperative care protocols can shed light on inflammation’s role in postoperative recovery, potentially guiding personalized treatment approaches. By integrating these advancements, the ERAS protocol can evolve to further enhance patient safety, expedite recovery, and solidify its role as a gold standard in AIS surgery.

## 4. Materials and Methods

### 4.1. Patients’ Population

A prospective pilot study was carried out at IRCCS Istituto Ortopedico Rizzoli between July 2021 and July 2022, following the approval of the Local Ethics Committee (CE AVEC 491/2021/Sper/IOR). The study was carried out in accordance with the principles of the Declaration of Helsinki. Thirty consecutive patients diagnosed with AIS undergoing PSF surgery, aged between 14 and 18, were enrolled after providing written informed consent. Blood samples were collected from each patient undergoing vertebral fusion both before and after surgery (2 days post-surgery). Exclusion criteria were patients with spinal infection, neoplastic diseases, cardiopathies, hypersensitivity to tranexamic acid, history of coagulopathy and/or previous thromboembolic events, or severe renal failure. We collected all data prospectively from our institution’s electronic medical records.

### 4.2. ERAS Pathway

**Figure 4:**
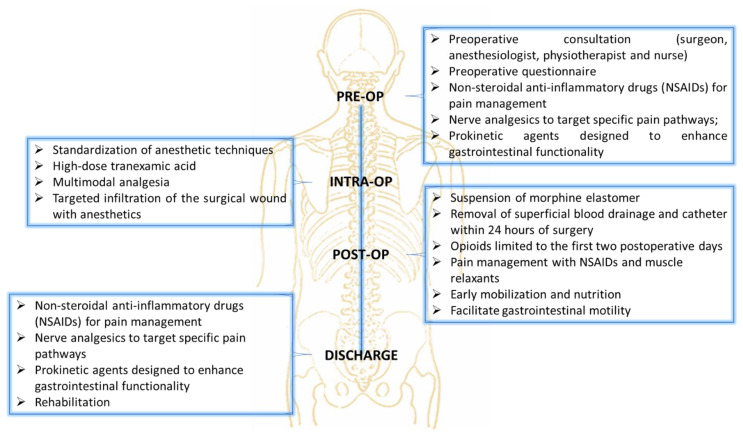
Schematic representation of ERAS pathway used in this study.

The ERAS pathway encompassed several strategies aimed at optimizing patient outcomes and minimizing complications (Figure 4). The care pathway begins with the patient and their family meeting the surgical team for a comprehensive orthopedic examination, which includes the Lenke classification and an assessment of the degree of deformity, such as the Cobb angle measurement. Following this, the patients consulted with the anesthesia team to initiate the ERAS care pathway. Preoperative measures also included the administration of medications such as non-steroidal anti-inflammatory drugs (NSAIDs) for pain management, nerve analgesics to target specific pain pathways (Gabpentin 300 mg), and prokinetic agents designed to enhance gastrointestinal functionality (Lactulose), which is often reduced following spinal correction surgery due to the impact on autonomic nervous system regulation and immobility. The intraoperative protocol emphasized the standardization of anesthetic techniques to improve consistency and outcomes. The anesthetic protocol was designed to ensure optimal hemodynamic stability, effective analgesia, and reduced intraoperative bleeding. Premedication included midazolam (3 mg IV) for anxiolysis. Induction was achieved using propofol (2–2.5 mg/kg IV) for hypnosis, fentanyl (1–2 µg/kg IV) for analgesia, and rocuronium (0.6–1 mg/kg IV) to facilitate tracheal intubation. Intraoperative analgesia was maintained with a continuous infusion of remifentanil (0.05–0.2 µg/kg/min), while sevoflurane (1–2 MAC) was used to maintain anesthesia. Tranexamic acid (1 ampoule, 500 mg IV) was administered prior to incision to minimize blood loss, with additional doses as needed during prolonged procedures. To manage inflammation and reduce postoperative nausea, dexamethasone (4–8 mg IV) was administered preoperatively. At the end of the procedure, morphine was used for postoperative analgesia, complemented by ketoprofen. To further mitigate postoperative blood loss, superficial blood drainage was employed for 24 h, replacing the traditional approach of deep subfascial drainage that is often left in place for over three days, thus reducing the risk of prolonged fluid accumulation and infection. Postoperative care focused on enhancing recovery through early mobilization of patients, which helps prevent complications such as thromboembolism, muscle deconditioning, and respiratory issues. The use of opioids was intentionally limited to the first two postoperative days to minimize the risk of side effects such as sedation, gastrointestinal dysfunction, and dependency. Pain management beyond this period relied on NSAIDs and muscle relaxants, which were tailored to address inflammation and prevent muscle spasms, which are common after spinal surgery. The approach was designed to balance effective pain control with the reduction of side effects, allowing patients to regain mobility and resume normal functions more quickly.

### 4.3. Surgical Procedure

The patient undergoes general anesthesia and neuromonitoring. They are positioned in the prone decubitus position on an Allen bed, with pillows used to protect bony prominences and to ensure proper intra-abdominal pressure. A sterile field is prepared using disposable drapes. The incision is made along the midline of the back, over the spinous processes. The surgeon then separates the muscles and soft tissues to expose the posterior elements of the spine. Using a freehand pedicle-finding technique, pedicle screws are inserted into the vertebrae, applying high-density instrumentation with a pair of pedicle screws at each level when possible. Once all the pedicle screws have been placed according to the preoperative plan, vertebral osteotomies are performed. Osteotomies are surgical procedures designed to correct skeletal deformities, including those caused by scoliosis. The most used types are the Smith-Petersen and Ponte osteotomies. Following this, one or more pre-shaped metal rods are inserted along the vertebral column and secured to the pedicle screws using nuts. These rods provide structural support and enable corrective maneuvers to realign spinal deformity. During the first phase of correction, rotational deformities are addressed using specialized instruments. In the subsequent phase, compressive and distraction forces are applied along the rods to straighten the spine, correct the scoliosis angle, and achieve proper balance in both the sagittal and frontal planes. To stabilize the spine and promote bone fusion between the vertebrae, bone grafts (autografts harvested from the patient during osteotomies) are utilized. In our series, non-autograft bone material was not utilized. At the end of the procedure, a suction drain is placed, and the surgical site is closed in layers.

### 4.4. Patient Demographic, Clinical Information, Radiographic and Surgical Data

Patient demographic, clinical, surgical, and radiographic data were collected prospectively. Demographic information included age, gender, age at menarche (for female patients), body mass index (BMI), and comorbidities. Blood parameters were evaluated both preoperatively and postoperatively (2 days post-surgery) and included White Blood Cell count, Red Blood Cell count, Hemoglobin, Hematocrit, Mean Corpuscular Volume (MCV), Mean Corpuscular Hemoglobin (MCH), Mean Corpuscular Hemoglobin Concentration (MCHC), Red Cell Distribution Width (RDW), Basophils, Neutrophils, Lymphocytes, Monocytes, Eosinophils, Platelet Count, Mean Platelet Volume (MPV), Prothrombin Activity Ratio, Prothrombin Activity, aPTT, Glucose, Creatinine and CRP. Additionally, pain intensity scores were documented preoperatively, postoperatively (1 and 2 days after surgery), and at discharge, using the VAS. Radiographic data included the preoperative and postoperative Cobb angle of the main curve, the correction rate of the main curve, and curve classification based on the Lenke system for AIS. Surgical details, such as the type of surgery performed, its duration, intraoperative blood transfusion, and the number of instrumented levels, were also evaluated. Postoperative outcomes, including complications, LOS, and rates of re-hospitalization, were analyzed.

### 4.5. Inflammatory Parameters

For serum preparation, pre- and post-surgery (2 days post-op) peripheral venous blood samples (~4–5 mL) were centrifugated at 2500 rpm for 15 min. Serum was aliquoted and stored at −80 °C until further testing. Serum IL-1α (lot. KE00268, Proteintech, Manchester, UK), IL-1β (lot. 20230328, Bio-Techne, Minneapolis, MN, USA), IL-6 (lot. 341574-010, Invitrogen, Waltham, MA, USA), IL-8 (lot. HUDC0064, PharmaGenie, Dublin, Ireland), IL-10 (lot. 40001589, Bio-Techne, Minneapolis, MN, USA), TNF-α (lot. 20230327, Bio-Techne, Minneapolis, MN, USA), and PGE2 (lot. L230505211, Cloud-Clone, Katy, TX, USA) were measured in triplicate using ELISA kits.

### 4.6. Statistical Analysis

Statistical analysis was performed using GraphPad Prism software (version 9.5.1). Data are presented as mean ± SD, with statistical significance set at *p* < 0.05. Normal distribution and homogeneity of variance were verified prior to analysis using the Shapiro–Wilk test. A two-way ANOVA with Bonferroni post-hoc correction was used for the analysis of VAS scores, while Student’s *t*-test with Bonferroni correction was applied for the evaluation of blood parameters and serum analyses to account for multiple comparisons and mitigate the risk of Type I errors.

## Figures and Tables

**Table 1:** Patient demographic characteristics.

ERAS Protocol (n = 30)	
Age (yrs)	15.33 ± 1.75
Gender (M: F)	4: 26
Age at menarche (for female patients)	12.40 ± 1.64
BMI (kg/m^2^)	21.61 ± 4.42
Nb levels fused	11.13 ± 2.06
Curve type (Lenke classification) (1:2:3:4:5:6)	8:2:6:0:3:3
Comorbidities	16.67%

**Table 2:** Patient clinical characteristics. (*) *p*  <  0.05, (**) *p*  <  0.005, (***) *p*  <  0.0005.

Blood Parameters and Reference Values	Pre-op (n = 30)	Post-op (n = 30)
White blood cells 3.6–10.5 10^9^/L	6.69 ± 0.95 ***	11.72 ± 3.23
Red blood cells 3.85–5.20 10^12^/L	4.74 ± 0.37	3.19 ± 0.46 ***
Hemoglobin 11.88–15.8 g/dL	13.40 ± 1.42	9.17 ± 1.29 ***
Hematocrit 35–45.5%	40.30 ± 2.71	27.23 ± 3.75 ***
MCV 80–101 fL	85.77 ± 3.09	85.62 ± 4.42
MCH, 27–34 pg	28.65 ± 1.53	28.96 ± 1.85
MCHC 31.5–36 g/dL	33.41 ± 0.89	33.84 ± 0.91
RDW %, 11.5–15%	12.52 ± 0.67	12.90 ± 0.90
RDW DV 39–51 fL	39.01 ± 2.30	40.06 ±1.82
Lymphocytes 1.1–4 10^9^/L	2.22 ± 0.49	1.28 ± 0.46 ***
Monocytes 0.1–0.9 10^9^/L	0.47 ± 0.15 ***	0.80 ± 0.26
Eosinophils 0.02–0.5 10^9^/L	0.21 ± 0.12	0.05 ± 0.05 ***
Basophils 0–0.2 10^9^/L	0.04 ± 0.02	0.02 ± 0.01 **
Neutrophils 1.5–7.7 10^9^/L	3.76 ± 0.95 ***	9.12 ± 2.57
Platelets count, 160–370 10^9^/L	293.62 ± 57.20	198.97 ± 46.10 ***
MPV, 8.5–11.5 fL	10.13 ± 0.62 *	10.63 ± 0.75
Prothrombic activity ratio <1.2	1.07 ± 0.05 ***	1.22 ± 0.07
Prothrombic activity INR <1.2	1.08 ± 0.06 ***	1.25 ± 0.09
aPTT ratio 0.82–1.25	1.15 ± 0.12 ***	1.36 ± 0.13
C-reactive protein <0.5 mg/dL	0.16 ± 0.25 ***	10.88 ± 4.62
Glucose 60–110 mg/dL	86.53 ± 10.14 ***	107.00 ± 7.75
Creatinine 0.5–1.20 mg/dL	0.66 ± 0.10	0.56 ± 0.16 *

**Table 3:** Perioperative outcomes and complications associated with the ERAS Protocol.

ERAS Protocol (n = 30)	
Duration (h)	3.50 ± 0.90
Intraoperative Blood Transfusion	20%
LOS (days)	6.97 ± 2.03
Re-Hospitalization	6.66%
Menageable Complications	20%

## Data Availability

The data presented in this study are available on request from the corresponding author.

## Abbreviations

The following abbreviations are used in this manuscript:

AIS	Adolescent idiopathic scoliosis
PSF	Posterior spinal fusion
ICU	Intensive Care Unit
ERAS	Enhanced Recovery After Surgery
TNF	Tumor necrosis factor
ILs	Interleukins
CRP	C-reactive protein
SIR	Systemic inflammatory response
VAS	Visual Analog Scale
LOS	Length of hospital stay
NSAIDs	Non-steroidal anti-inflammatory drugs
BMI	Body mass index
MCV	Mean Corpuscular Volume
MCH	Mean Corpuscular Hemoglobin
MCHC	Mean Corpuscular Hemoglobin Concentration
RDW	Red Cell Distribution Width
MPV	Mean Platelet Volume
aPTT	Activated Partial Thromboplastin Time
SD	Standard deviation
PGE2	Prostaglandin E2
